# The relationship between inflammatory factors and heart failure: evidence based on bidirectional Mendelian randomization analysis

**DOI:** 10.3389/fcvm.2024.1378327

**Published:** 2024-12-12

**Authors:** Xuanchun Huang, Lanshuo Hu, Jun Li, Shiyi Tao, Tiantian Xue

**Affiliations:** ^1^ Cardiology Department, Guang'anmen Hospital, China Academy of Traditional Chinese Medicine, Beijing, China; ^2^ Institute of Digestive Diseases, Xiyuan Hospital, China Academy of Traditional Chinese Medicine, Beijing, China

**Keywords:** Inflammatory factors, Mendelian randomization, heart failure, genetics, causal association

## Abstract

**Objective:**

Inflammatory factors play a crucial role in the onset and progression of heart failure. To further explore the causal relationship between inflammatory factors and heart failure, we employed bidirectional Mendelian randomization analysis to investigate the causal links between 91 inflammatory cytokines and heart failure.

**Methods:**

We conducted our study using the bidirectional Mendelian randomization approach. Data on 91 inflammatory factors were sourced from large-scale public genome-wide association study databases, while heart failure data were obtained from the FINNGEN database. The relationships between inflammatory factors and heart failure were evaluated using five methods: MR-Egger regression model, Inverse Variance Weighted method, Simple mode model, Weighted mode model, and Weighted median. Results were subjected to FDR multiple testing correction, and significant findings were discussed in detail. To enhance the robustness of our findings, various sensitivity analyses were conducted, including MR Egger intercept, MR-PRESSO and Cochran Q test.

**Results:**

Our forward Mendelian randomization study indicated that, of the 91 inflammatory factors examined, seven showed a causal relationship with heart failure. Four of these factors were significantly causally related to the incidence of heart failure: CXCL9 and IFN-γ as promotive factors, and LIFR and UPA as potential protective factors. Three inflammatory factors had a potential causal relationship with heart failure, with DNER as a potential protective factor, and MMP-1 and CD6 as potential promotive factors. Reverse Mendelian randomization suggested that the onset of heart failure might potentially influence the levels of four inflammatory factors, with ARTN and FGF5 decreasing after the onset of heart failure, and SLAM and MMP-10 increasing. Additionally, reliability tests of this Mendelian randomization, including MR-Egger intercept and MR-PRESSO tests, revealed no evidence of pleiotropy, and Cochran's Q test also confirmed the reliability of our results.

**Conclusion:**

We identified CXCL9, IFN-γ, LIFR, and UPA as potential inflammatory factors associated with heart failure through forward Mendelian randomization. These findings suggest potential targets but require further validation.

## 1 Introduction

The immune response and ongoing inflammation in the heart are key factors in triggering ventricular remodeling and heart function decline, directly or indirectly contributing to the development of heart failure. Early successes in cardiovascular clinical trials with Canakinumab (anti-Interleukin-1 beta), Ziltivekimab (anti-Interleukin-6), and Colchicine have solidified the approach of targeting inflammation to reduce cardiovascular risk (1). However, the previous optimism for Tumor Necrosis Factor-Alpha (TNF-α) targeted therapy, which ultimately failed, suggests that some cytokine-based approaches may have limited roles in the treatment of heart failure (2). Therefore, it is necessary to identify other cytokines that may serve as new targets in cardiovascular conditions.

Previous studies on cytokines in heart failure have mainly focused on various inflammatory cytokines, such as Interleukin (IL), Interferon (IFN), Tumor Necrosis Factor superfamily (TNF), Colony Stimulating Factor (CSF), Chemokine Family(CF), and Growth Factor (GF) (3). These inflammatory cytokines play critical roles in immune responses, inflammatory reactions, and tissue repair during heart failure. Some cytokines have already been preliminarily validated for their roles, such as the previously mentioned TNF-α, and related cytokines like IL-6, IL-1β, IL-8, IL-18 (4–7), which may promote the onset of heart failure. In contrast, some cytokines like IL-10, IL-37, IL-35 (8–10), delay ventricular remodeling and alleviate the progression of heart failure by inhibiting inflammatory responses. The imbalance of these inflammatory cytokines can cause endogenous stress damage to the myocardium, affect myocardial contractility, induce myocardial apoptosis, and further activate Matrix Metalloproteinases (MMP) and collagen formation, leading to extracellular matrix degradation and fibrotic scar formation, ultimately impairing heart function and promoting the onset of heart failure. Conversely, the development of heart failure may also cause fluctuations in some inflammatory cytokines and lead to new pathophysiological mechanism changes, such as the recent approval by the US Food and Drug Administration (FDA) of two prognostic inflammatory biomarkers, soluble Suppression of Tumorigenicity 2 (ST2) and Galectin-3 (11, 12). The approval of these two cytokines further promotes in-depth research into the relationship between inflammatory cytokines and heart failure. However, the complex pathophysiological network between inflammatory cytokines and heart failure has not yet been fully constructed.

As mentioned earlier, the inflammatory response plays a crucial role in the progression of heart failure, and the level of inflammatory cytokines is also an important indicator for judging the severity and prognosis of heart failure. A deep understanding of the relationship between inflammatory cytokines and heart failure, and clarifying their mechanisms of action, is of great significance for the prevention and treatment of heart failure and provides direction and basis for the discovery of new drug targets (13).

In recent years, due to genome-wide association studies (GWAS) and the public availability of various data resources, it is convenient to use Mendelian randomization to analyze the causal relationship between different exposures and outcomes, thus excluding the influence of various confounders and confounding factors. Therefore, this article intends to explore the causal relationship between circulating inflammatory cytokines and heart failure using bidirectional Mendelian randomization, to predict and verify the role of inflammatory cytokines in the progression of heart failure, providing evidence for treating and preventing diseases from the perspective of inflammation.

## 2 Analytical methods and data sources

### 2.1 Study design

**Figure 1:**
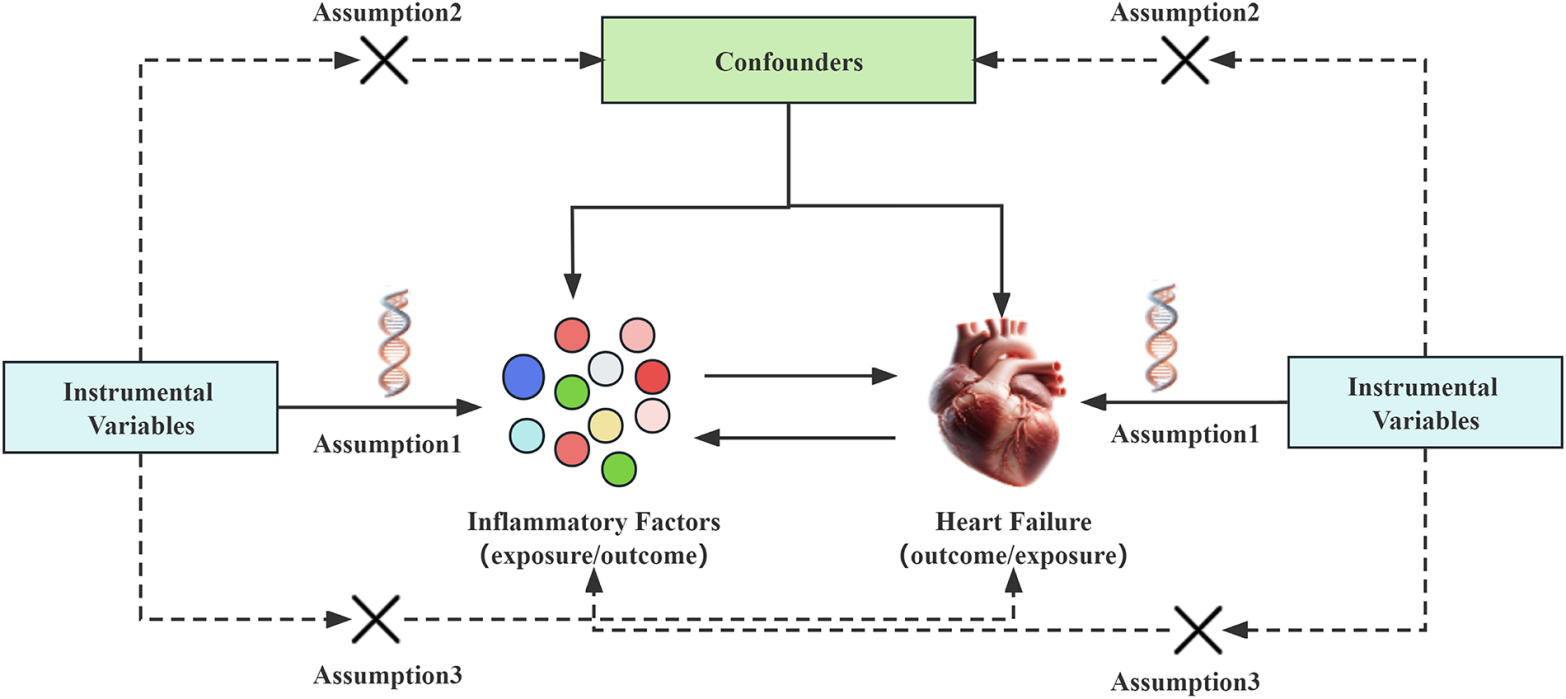
Mendelian randomization studies hypothesize that genetic variations are only related to exposure. The instrumental variable affects the outcome of heart failure through inflammatory factors, rather than affecting the outcome of heart failure through confounding factors or other causal pathways. Conversely, the instrumental variable can only affect the levels of inflammatory factors through heart failure, and not through confounding factors or other pathways.

In Mendelian randomization analysis, the instrumental variables need to satisfy three assumptions (14): (I) The instrumental variable must be strongly associated with the exposure factor; (II) The instrumental variable should not be associated with confounding factors; (III) The instrumental variable must not be directly related to the outcome event, and its effect should only occur through the exposure factor. This means that in the forward Mendelian randomization analysis, the chosen instrumental variables can only be related to and affect the occurrence of heart failure through the inflammatory factors in the blood, and not through confounding factors or by directly influencing the onset of heart failure. Conversely, in the reverse Mendelian randomization analysis, the selected instrumental variables can only affect the levels of inflammatory factors through heart failure, and not directly impact the levels of inflammatory factors see Figure 1.

### 2.2 Date source

Our data on 91 inflammatory factors were derived from an article titled “Genetics of circulating inflammatory proteins identifies drivers of immune-mediated disease risk and therapeutic targets” published in September 2023 in the journal “Nature Immunology” (15). This study was conducted by the SCALLOP consortium and involved 14,824 European participants. Additionally, our heart failure GWAS data came from the R10 version of the FINNGEN database, utilizing heart failure data categorized under ICD codes I11.0, I13.0, I13.2, I50. This dataset comprised 412,181 participants, including 29,672 in the case group and 382,509 in the control group. It is important to note that our data included patients identified as having the primary disease or solely suffering from a certain disease (admission, discharge, death), thus our study did not encompass heart failure due to congenital heart disease, genetic heart diseases, or cardiomyopathies (such as dilated cardiomyopathy, or other congenital cardiac structural abnormalities). Additionally, our heart failure data did not differentiate between heart failure with preserved ejection fraction, and heart failure with mildly reduced ejection fraction, as indicated by some of the ICD codes.

When the strictest criteria were applied to limit the data on inflammatory factors, the number of single nucleotide polymorphism (SNP) related to inflammatory factors was too few. Therefore, in our study, when choosing 91 types of inflammatory factors as the exposure factor, the *P*-value for SNPs was set at <5 × 10^−6^. When selecting heart failure as the exposure factor, as the number of SNPs was sufficient, we required the *P*-value for SNPs to be <5 × 10^−8^. To increase the credibility of the study, we excluded SNPs with linkage disequilibrium, setting the linkage disequilibrium coefficient *r*^2^ to 0.001, and defining the linkage disequilibrium region width as 10,000 kb. Missing SNPs were replaced with SNPs that had a high linkage with them, and palindromic sites were deleted.

Two-sample Mendelian randomization analysis requires that the two samples be independent of each other and come from different populations with similar gender, age, and racial characteristics. Therefore, the population in this study consisted of Europeans, and different databases were used for analysis. Since the information used in this article is publicly published, there are no ethical controversies in this study.

## 3 Statistical analysis

To better assess the strength of the association between instrumental variables and exposure factors, we used the calculation of *F*-statistics to screen for weak instrumental variables. It is generally considered that if the *F*-value of an SNP instrumental variable is less than 10, there may be a weak association of the instrumental variable, affecting the results of Mendelian randomization analysis. Therefore, in this study, instrumental variables with an *F*-value less than 10 were excluded (see Supplementary Tables S1, S2 for details). The formula chosen for the calculation was the standard method for *F*-statistics calculation (16).

Statistical analysis is the core part of Mendelian randomization analysis. In this phase, we used MR-Egger regression model (MR-Egger), Inverse Variance Weighted method (IVW), Simple mode model, Weighted mode model, and Weighted median method for assessment (17). For detecting pleiotropy, we used MR-Egger intercept test and MR-PRESSO test (18), and compared the MR analysis results before and after correcting for horizontal pleiotropy outliers. If a significant difference was found in the statistical values before and after correction, we excluded the outlier SNPs.

The presence of heterogeneity can affect the robustness of the analysis. Therefore, we used the Cochran *Q* test (19) to assess the heterogeneity of the included SNPs. If *P* < 0.05, it was considered that there was heterogeneity in the study, and for data with heterogeneity, the IVW random model was used for analysis; otherwise, the IVW fixed model was employed. To further confirm the impact of each SNP on the overall results, we also conducted a “Leave-One-Out” analysis to observe the impact of individual SNPs on the results. If the “Leave-One-Out” analysis results were inconsistent with the causal effect analysis results, it indicated the presence of non-specific SNPs, which could affect the causal estimation effect. This method was performed using the Two Sample MR package in *R* software, with a significance level of *α* *=* 0.05.

For the results after Mendelian analysis, we applied FDR (20) multiple comparisons correction. Results with *P* < 0.05 but not satisfying the FDR test were considered as suggestive evidence of a potential causal relationship, indicating the need for further exploration and verification in the future. Results positive for FDR were considered strong positive results, affirming their causal relationship.

## 4 Result

### 4.1 Causality and sensitivity analysis of forward Mendelian randomization analysis

**Figure 2:**
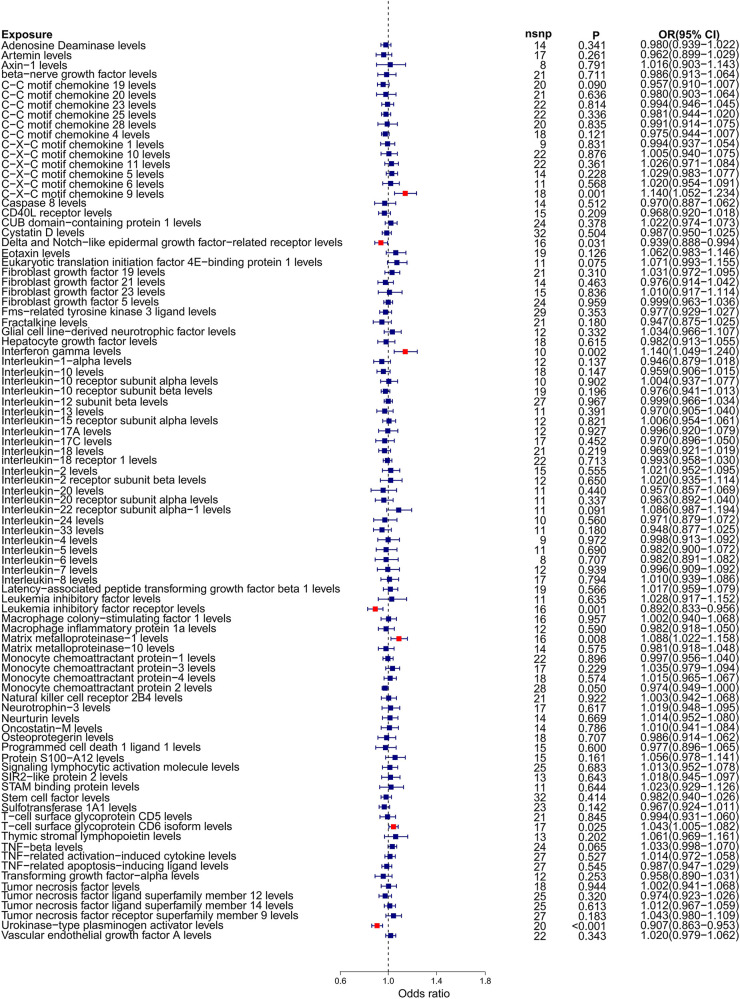
Forest plot of Mendelian randomization analysis with 91 inflammatory factors as exposure and heart failure as outcome.

**Figure 3:**
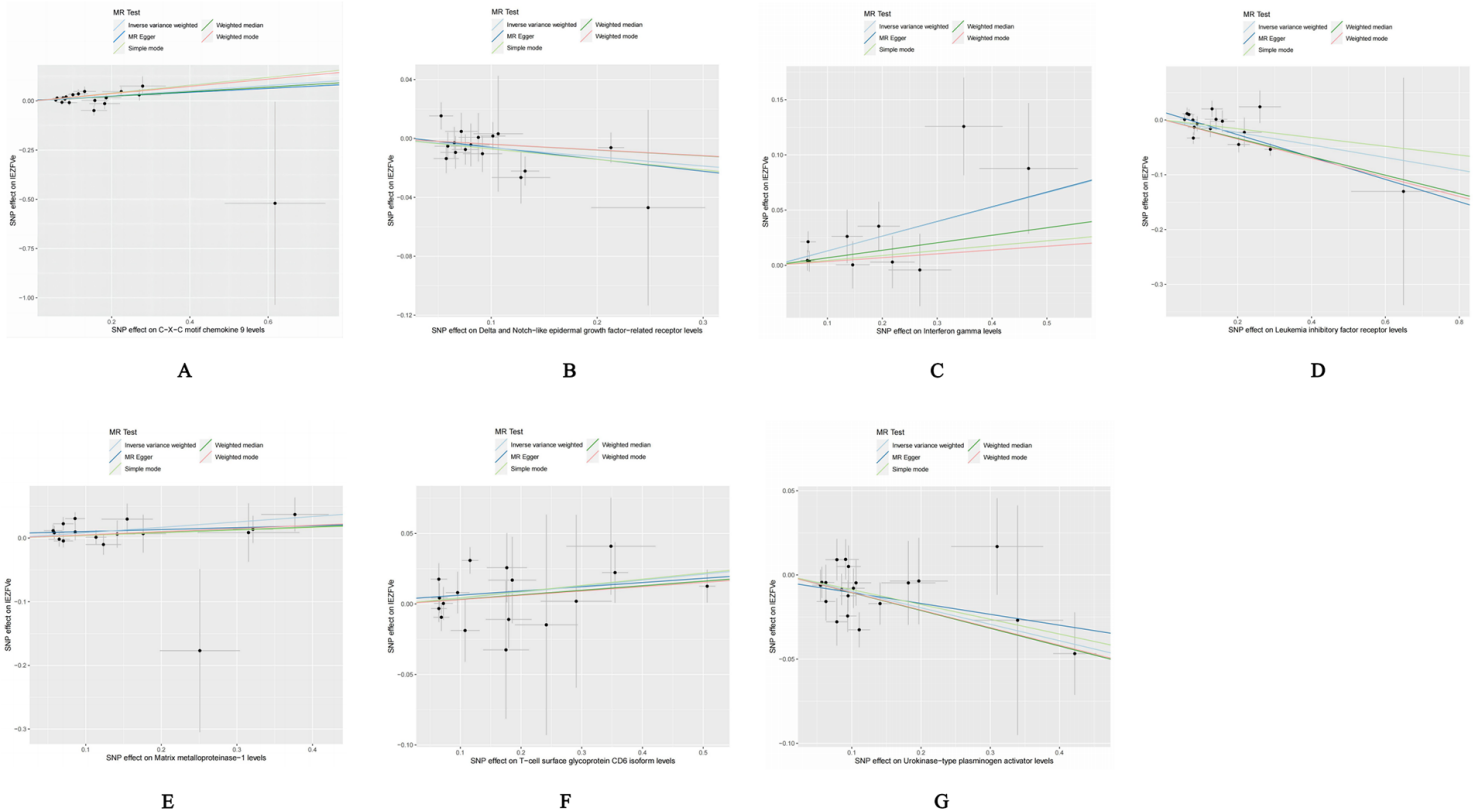
Scatter plot of positive results after cis-Mendelian randomization analysis: **(A)** CXCL9-HF **(B)** DNER-HF **(C)** IFNγ-HF **(D)** LIFR-HF **(E)** MMP1-HF **(F)** CD6-HF **(G)** UPA-HF.

Our forward Mendelian randomization study, using 91 inflammatory factors as exposures, identified causal relationships between seven inflammatory factors and heart failure. Among these, four have a significant causal relationship with the onset of heart failure, and three are potential causal factors. Significant causal relationship factors: C-X-C motif chemokine 9 (CXCL9, OR = 1.140, 95% CI = 1.052–1.234, *P* = 0.001, *P*_FDR_ = 0.005); Interferon-gamma (IFN-γ, OR = 1.140, 95% CI = 1.049–1.240, *P* = 0.002, *P*_FDR_ = 0.0363); Leukemia Inhibitory Factor Receptor (LIFR, OR = 0.892, 95% CI = 0.833–0.956, *P* = 0.001, *P*_FDR_ = 0.0004); Urokinase-type Plasminogen Activator (UPA, OR = 0.907, 95% CI = 0.863–0.953, *P* = 0.0001, *P*_FDR_ = 0.005); In these, CXCL9 and IFN-γ are identified as risk factors promoting heart failure, whereas LIFR and UPA are protective factors against heart failure. Potential causal relationship factors: Delta/Notch-like EGF repeat containing receptor (DNER, OR = 0.939, 95% CI = 0.888–0.994, *P* = 0.031, *P*_FDR_ = 0.382); Matrix Metallopeptidase 1 (MMP-1, OR = 1.088, 95% CI = 1.022–1.158, *P* = 0.008, *P*_FDR_ = 0.095); T-cell surface glycoprotein CD6 isoform (CD6, OR = 1.043, 95% CI = 1.005–1.082, *P* = 0.002, *P*_FDR_ = 0.348). Here, DNER is a potential protective factor against heart failure, while MMP-1 and CD6 are potential risk factors. The relationships of other inflammatory factors with heart failure are illustrated in Figure 2. Additionally, scatter plots for positive results were generated as shown in Figure 3.

To ensure the credibility of our results, we conducted various statistical analyses (as shown in Table 1). The MR-Egger regression intercepts were close to zero with *P*-values greater than 0.05, indicating no horizontal pleiotropy in the forward Mendelian randomization. Similarly, MR-PRESSO tests showed *P*-values greater than 0.05, indicating no SNPs with horizontal pleiotropy and no outlier SNPs. The Cochran *Q* tests revealed *P*-values greater than 0.05 for all groups, suggesting no heterogeneity in the study results. “Leave-One-Out” analysis on the inverse-variance weighted results showed no significant changes after the sequential removal of each SNP, aligning with the causal effect analysis results and indicating the reliability of our analysis (as seen in Supplementary Figure S1). Funnel plots also demonstrated an even distribution of genes on both sides of the *β* value, similar to the pattern in the scatter plots, indicating no apparent gene bias (as seen in Supplementary Figure S2).

**Table 1:** Sensitivity and stability analysis of forward Mendelian randomization.

Information		MR-Egger intercept			CochranQ		MR-PRESSO
Exposure	FDR	SE	Intercept	*P*	CochranQ	*P*	Global *P*
CXCL9	0.005	0.010	0.004	0.725	23.449	0.135	0.142
DNER	0.382	0.007	0.002	0.756	10.982	0.754	0.711
IFN-γ	0.036	0.009	0.000	0.979	9.529	0.390	0.435
LIFR	0.000	0.008	0.014	0.112	29.548	0.214	0.118
MMP-1	0.095	0.007	0.007	0.284	16.615	0.342	0.376
CD6	0.348	0.005	0.003	0.541	16.379	0.427	0.446
UPA	0.005	0.006	−0.004	0.496	19.108	0.450	0.471

### 4.2 Causality and sensitivity analysis of inverse Mendelian randomization analysis

**Figure 4:**
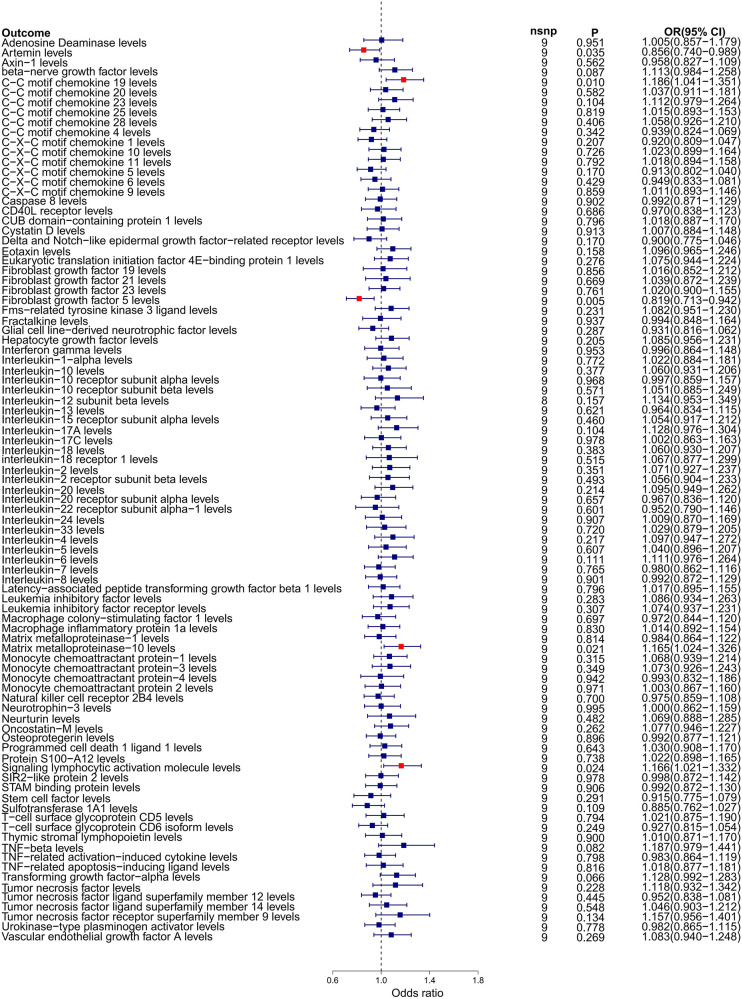
Forest plot of Mendelian randomization analysis with 91 inflammatory factors as outcome and heart failure as exposure.

**Figure 5:**
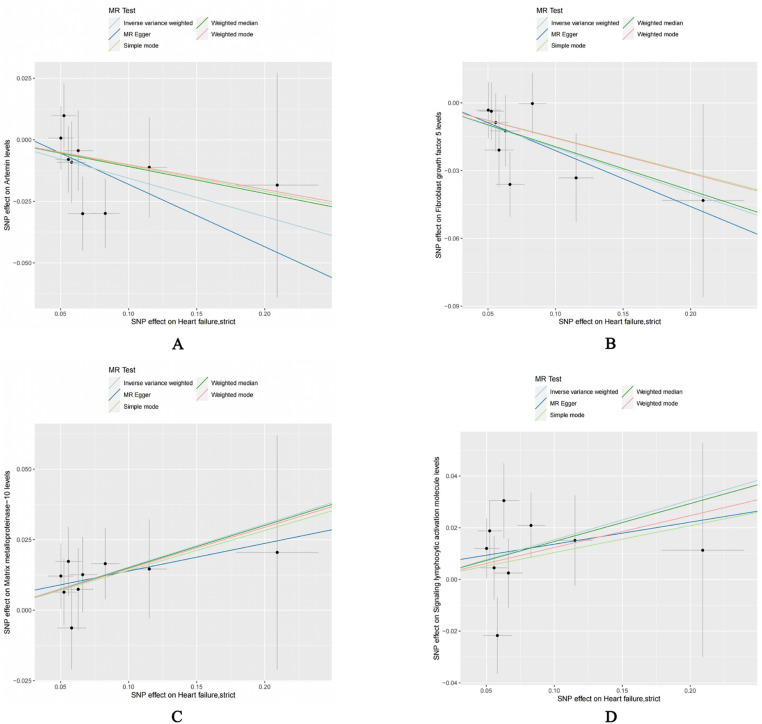
Scatterplot of positive results after inverse Mendelian randomization analysis: **(A)** HF-ARTN **(B)** HF-FGF5 **(C)** HF-MMP10 **(D)** HF-SLAM.

Our reverse Mendelian randomization study, considering heart failure as the exposure and 91 inflammatory factors as outcomes, identified correlations with five inflammatory factors based on the IVW method with *P* < 0.05. However, the results for Chemokine Ligand 19 (CCL19) were excluded due to contradictory slopes between the MR-Egger and IVW methods, indicating CCL19's datas non-compliance with the assumptions of Mendelian randomization. Therefore, we primarily focused on the other four inflammatory factors potentially influenced by heart failure, as detailed below: Artemin (ARTN, OR = 0.856, 95% CI = 0.740–0.989, *P* = 0.035, *P*_FDR_ = 0.532); Fibroblast Growth Factor 5 (FGF5, OR = 0.819, 95% CI = 0.713–0.942, *P* = 0.005, *P*_FDR_ = 0.398); Matrix Metallopeptidase 10 (MMP-10, OR = 1.165, 95% CI = 1.024–1.326, *P* = 0.021, *P*_FDR_ = 0.401); Signaling Lymphocytic Activation Molecule (SLAM, OR = 1.166, 95% CI = 1.021–1.332, *P* = 0.024, *P*_FDR_ = 0.401). The relationships between heart failure and other inflammatory factors are illustrated in Figure 4. Additionally, scatter plots for positive results were generated as shown in Figure 5. The absence of FDR-positive results in reverse Mendelian randomization suggests that the impact of heart failure on these inflammatory factors is potentially causal, warranting further investigation. There were no common inflammatory factors between the reverse and forward Mendelian randomization, eliminating the possibility of bidirectional causality.

Similar to our forward analysis, we conducted various statistical analyses for reverse Mendelian randomization to reinforce the sensitivity and stability of our results (see Table 2). The MR-Egger and MR-PRESSO tests showed *P*-values >0.05, indicating no horizontal pleiotropy. The Cochran *Q* test results demonstrated no heterogeneity in the study. Finally, the Leave-One-Out analysis results were reliable (as seen in Supplementary Figure S3), and funnel plots showed an even distribution of genes (as seen in Supplementary Figure S4), confirming the reliability of our reverse Mendelian randomization results.

**Table 2:** Sensitivity and stability analysis of reverse Mendelian randomization.

Information		MR-Egger intercept			CochranQ		MR-PRESSO
Outcome	FDR	SE	Intercept	*P*	CochranQ	*P*	Global *P*
ARTN	0.532	0.015	0.007	0.653	5.812	0.668	0.668
FGF5	0.398	0.015	0.004	0.814	5.227	0.733	0.744
MMP-10	0.401	0.014	0.004	0.777	2.006	0.981	0.986
SLAM	0.401	0.015	0.005	0.746	8.510	0.385	0.447

## 5 Discussion

In this study, we utilized a bidirectional two-sample Mendelian randomization method to systematically assess the causal effects of 91 circulating inflammatory factors on heart failure. We discovered that 7 out of these 91 inflammatory factors influence heart failure, with 4 showing a significant causal relationship (CXCL9, IFN-γ, LIFR, UPA) and 3 being potentially related influencing factors (DNER, MMP-1, CD6). These factors may play a role in the development and progression of heart failure, and intervening in them could be beneficial in the treatment of heart failure.Additionally, our reverse Mendelian randomization study indicated that heart failure might lead to fluctuations in the levels of four inflammatory factors (ARTN, FGF5, MMP-10, SLAM), which could potentially serve as biomarkers for predicting heart failure in the future. We will now focus on discussing the inflammatory factors with a significant causal relationship.

CXCL9, a member of the CXC chemokine family, is a small molecule protein typically secreted by immune and endothelial cells during inflammation or immune responses. This chemokine facilitates the transformation of T cells into Th1/Th17 cells by binding to its specific receptor, CXCR3, thereby promoting inflammation (21). CXCL9 can also amplify the inflammatory cascade by recruiting additional macrophages and neutrophils, to the site of injury, further inducing the activation of endothelial cells and fibroblasts, contributing to vascular dysfunction and myocardial remodeling (22). In cardiovascular diseases, especially heart failure, the role of CXCL9 may lead to further damage and fibrosis in cardiac tissue, exacerbating heart failure. Specifically, CXCL9 has been implicated in promoting cardiac fibrosis by stimulating fibroblast proliferation and extracellular matrix deposition, processes closely linked to cardiac stiffness and diastolic dysfunction. Additionally, CXCL9 may interact with oxidative stress pathways and pro-apoptotic signals, accelerating cardiomyocyte loss. This hypothesis is supported by evidence from both animal experiments and clinical studies. Altara R observed increased expression of CXCL9 in both distant and near-infarct areas of the heart in rats, maintaining high levels for up to 16 weeks post-myocardial infarction, suggesting a continuous role of CXCL9 in post-infarction heart failure (23). Moreover, they (24) also found elevated levels of CXCL9 in circulation in rat models of hypertensive myocardial hypertrophy or compensatory myocardial hypertrophy due to heart failure following myocardial infarction. Furthermore, in a cohort study involving patients with hypertension combined with heart failure and a healthy control group, they (25) reported higher levels of CXCR3 ligands, including CXCL9, in patients with left ventricular diastolic dysfunction. Additionally, they used the levels of CXCL9, CXCL10, and CXCL11 in their study to adjust the prediction in the heart failure model, finding that including CXCL9, CXCL10, and CXCL11 improved the prognosis prediction of heart failure (26). Overall, there is substantial evidence linking increased circulating CXCL9 to the development of heart failure, and our Mendelian study further supports this perspective.

IFN-γ is a pleiotropic cytokine primarily secreted by Th1 cells, natural killer (NK) cells, and macrophages. It exerts its effects by binding to the IFN-γ receptor, activating the JAK-STAT signaling pathway. This signaling cascade can regulate numerous processes, including immune cell activation, fibrosis, and cardiomyocyte function. However, its role in heart failure is context-dependent, as both detrimental and adaptive functions have been observed in different experimental and clinical settings. Our Mendelian randomization further supports a positive role for IFN-γ in heart failure, as do the following pieces of evidence. In a mouse model of hypertension-induced heart failure, Levick SP et al. found significantly elevated myocardial IFN-γ levels in 20-week-old animals, an age at which hypertrophy and fibrosis are present (27). Yu Q and colleagues observed in mice that inducing the Th1T-cell phenotype led to a 12-fold increase in cardiac IFN-γ levels, associated with cardiac hypertrophy, increased total collagen, extensive collagen cross-linking, and left ventricular stiffening (28). These findings suggest that IFN-γ may exacerbate adverse cardiac remodeling by promoting fibroblast activation, increasing extracellular matrix deposition, and enhancing collagen cross-linking, which reduces myocardial compliance and contributes to diastolic dysfunction. Additionally, IFN-γ-driven inflammation may activate endothelial cells and induce a pro-thrombotic state, further compromising cardiac microvascular function. Han and others (29) reported that infusion of angiotensin II in wild-type mice resulted in increased cardiac IFN-γ levels. In mice with a knockout of the IFN-γ gene, despite similar levels of cardiac hypertrophy to wild-type mice, there was a significant reduction in cardiac fibrosis. This highlights a pivotal role for IFN-γ in mediating fibrotic pathways independently of hypertrophy. It suggests that targeting IFN-γ or its downstream signaling may selectively mitigate fibrosis while preserving necessary hypertrophic responses during early cardiac adaptation. *in vitro* experiments (30) showed that IFN-γ has a direct effect on cardiac myocytes; studies using isolated rat atria indicated that IFN-γ concentrations between 2 and 10 U/ml have an inhibitory effect on contraction. Stimulation of mouse fetal cardiomyocytes with IFN-γ led to a 15-fold increase in atrial natriuretic factor (ANF) expression, suggesting a potential exacerbating role for IFN-γ in heart failure (31). Mechanistically, the inhibitory effects on contraction may be linked to IFN-γ-mediated alterations in calcium handling and reduced sarcoplasmic reticulum function. Furthermore, elevated ANF expression suggests a maladaptive stress response, contributing to volume overload and heart failure progression. Clinical trials also highlight the role of IFN-γ. A study involving 72 patients with congestive heart failure secondary to non-ischemic diseases (48 with dilated cardiomyopathy, 24 with hypertensive heart disease), none of whom were treated with statins, found elevated serum IFN-γ levels (92.69 vs. 66.41 pg/ml) compared to controls (32). This association between elevated IFN-γ levels and heart failure severity suggests its potential as a biomarker for disease progression. Our research builds on existing studies to reinforce the evidence of IFN-γ's role in the progression of heart failure, but more experiments are needed in the future to validate this perspective.

The LIF/LIFR signaling pathway plays a crucial role in myocardial protection, particularly under stress conditions such as ischemia or hemodynamic overload. LIFR, as the receptor for leukemia inhibitory factor (LIF), is often co-expressed with its ligand, enabling efficient signal transduction. When LIF binds to LIFR, it activates downstream pathways such as PI3K/AKT, JAK-STAT, and MAPK, which are associated with anti-apoptotic and anti-inflammatory responses. The cardioprotective role of LIF and LIFR has been demonstrated in several experimental models. Miyamoto S and colleagues showed that under ischemic conditions, the binding of LIF to LIFR promotes mitochondrial AKT protein phosphorylation, increases the binding of hexokinase II to mitochondrial AKT, prevents mitochondrial peroxidation, and reduces the opening of the mitochondrial permeability transition pore (mPTP) membrane channel (33). This mechanism not only mitigates oxidative stress during ischemic injury but also supports mitochondrial energy homeostasis, contributing to the long-term survival and function of cardiomyocytes. Berry MF, using an adenovirus to overexpress the LIF gene in a rat myocardial infarction model, found that rats overexpressing LIF had more surviving myocardium and less fibrosis, suggesting a cardioprotective effect of LIF binding to its receptor (34). Notably, this study also demonstrated that LIF promotes angiogenesis and suppresses inflammation in the peri-infarct region, thereby improving post-infarction ventricular remodeling. Additionally, Wang F and colleagues found that hemodynamic overload can increase LIF expression in the heart, thereby protecting the heart from failure by blocking apoptosis and stimulating cardiac hypertrophy. However, the researchers also noted that LIF does not have a significant impact on myocardial contractility (35). This indicates that LIF's cardioprotective role is primarily mediated through structural and metabolic regulation rather than direct modulation of contractile function. Further studies suggest that LIF's regulation of extracellular matrix homeostasis is a key mechanism in delaying fibrosis and preserving cardiac function. As the ligand for LIF, LIFR expression may be significantly upregulated in certain stress or disease states, such as myocardial infarction, as a protective and reparative mechanism of the body. The overexpression of LIFR may amplify the sensitivity to LIF, enhancing the activation of signaling pathways to provide more effective cardiac protection. Additionally, high LIFR expression is closely linked to the survival and differentiation of cardiac stem cells, suggesting a role for LIFR in cardiac regeneration. Thus, previous research has confirmed the cardioprotective role of the LIF/LIFR pathway (36), and our Mendelian randomization study supports this finding.

UPA is one of the ligands for urokinase-type plasminogen activator receptor (UPAR), and together they play a crucial role in the progression of cardiovascular diseases. They are key regulators of signaling functions that influence cell behavior, including adhesion, surface protein hydrolysis, migration, proliferation, chemotaxis, and extravasation. Beyond their established roles in cellular dynamics, UPA and UPAR are increasingly recognized for their dual involvement in tissue repair and pathological remodeling, making them significant players in both homeostasis and disease progression. Traditionally, UPA is known for converting plasminogen into plasmin, facilitating thrombolysis and tissue healing processes. However, recent experimental studies have highlighted the roles of UPA and UPAR in the progression of heart failure (37–40). Dergilev K and colleagues (37) studied UPAR gene knockout in mice and observed a reduction in interstitial cardiac stem cells, which impaired cardiac repair capabilities and increased myocardial apoptosis. These findings suggest that UPAR supports cardiac regeneration by maintaining a viable pool of cardiac stem cells, a critical factor for long-term myocardial integrity under stress conditions. The protective effects of UPA have also been demonstrated in other diseases. Horowitz J.C. and colleagues found that inducing UPA attenuated pulmonary fibrosis in a mouse model (41), while Sun C. showed that UPA gene therapy alleviated liver fibrosis in a rat model (42). These results suggest that UPA might prevent heart failure through anti-fibrotic mechanisms or by protecting myocardium from apoptosis, aligning with Mendelian randomization studies that support a protective role of UPA against heart failure. This anti-fibrotic effect could involve direct modulation of extracellular matrix turnover and inhibition of myofibroblast activation, mechanisms that are crucial in preventing adverse cardiac remodeling. However, some evidence suggests that UPA and UPAR may serve as prognostic factors in heart failure. Victor J. and colleagues (43) reported that elevated UPA and UPAR levels are associated with myocardial infarction, dilated cardiomyopathy, cardiac fibrosis, and heart failure. Heymans S. and colleagues (44) demonstrated that UPA inhibition attenuated left ventricular remodeling and dysfunction after acute pressure overload in mice, indicating UPA's role in pathological remodeling. Furthermore, Heymans S. found that inhibition of plasminogen activators or matrix metalloproteinases prevents cardiac rupture but impairs therapeutic angiogenesis and causes cardiac failure, suggesting their complex roles in cardiac remodeling post-infarction (45). This discrepancy highlights the complexity of UPA/UPAR signaling, which appears to exhibit context-dependent effects that vary with disease stage, tissue environment, and systemic factors. Further studies are needed to delineate these dual roles and clarify the conditions under which UPA promotes repair vs. remodeling. In addition, our Mendelian randomization also identified potential causative inflammatory markers such as MMP-1 and T-CD6, which may promote the onset of heart failure, and DNER, which may alleviate it. However, since these factors did not pass the FDR test, even though many studies have shown their relationship with heart failure (46–48), we have not discussed them in depth.

Moreover, reverse Mendelian randomization analysis identified inflammatory factors such as ARTN, FGF5, MMP-10, and SLAM, whose increased secretion may represent an adaptive response to heart failure. However, as these factors did not pass the FDR test, we discuss them briefly. The release of these factors may be induced by the persistent mechanical stress, ischemia-reperfusion injury, and chronic inflammation commonly associated with heart failure. While these factors may initially protect myocardial tissue or aid in repair, their sustained elevation often drives pathological progression. Specifically, elevated ARTN levels may represent a compensatory mechanism of the myocardium to adapt to excessive sympathetic nervous system activation. Although ARTN promotes sympathetic nerve fiber regeneration and branching, aiding neural recovery in the short term, prolonged elevation may exacerbate arrhythmias and ventricular dysfunction (49). Similarly, increased FGF5 secretion might improve the myocardial environment by promoting angiogenesis and tissue repair, but its persistent elevation could activate fibroblasts, leading to excessive extracellular matrix deposition, myocardial fibrosis, and chronic low-grade inflammation, worsening cardiac dysfunction (50). Elevated MMP-10 levels reflect the myocardium's need to regulate extracellular matrix dynamics in heart failure. While it initially helps clear damaged extracellular matrix components, prolonged overexpression disrupts extracellular matrix structural integrity, causing ventricular dilation and impaired contractility (51). Furthermore, increased SLAM levels signal immune system overactivation triggered by heart failure. Although this activation initially aids in clearing necrotic cells, chronic immune responses exacerbate myocardial inflammation and damage, impairing myocardial repair by influencing cell adhesion and apoptosis (52). The elevation of these inflammatory factors not only results from the pathological environment of heart failure but also actively contributes to its progression by amplifying chronic inflammation, disrupting immune regulation, and causing imbalances in the extracellular matrix. This creates a feedback loop that accelerates disease advancement. Further research into the regulatory mechanisms and roles of these factors could provide critical insights into the complex pathology of heart failure and guide the development of novel therapeutic strategies.

In summary, our study has several advantages. We utilized 91 inflammatory factors released by the SCALLOP consortium, covering essentially all known inflammatory factors, making our study comprehensive. Secondly, the results of reverse Mendelian randomization showed no intersection with the inflammatory factors from the forward Mendelian randomization, indicating no bidirectional causality among these inflammatory factors, which makes our results quite reliable. Lastly, we conducted rigorous Mendelian randomization analysis and identified several inflammatory factors that may cause heart failure, which after multiple tests showed positive significance, potentially offering unique value for the future prevention and treatment of heart failure.

Finally, we must acknowledge the limitations of our study. Indeed, some inflammatory factors that are more certain in animal and cell studies, such as TNF-α and IL-6, showed no statistical significance in our study. We admit that using only the FINNGEN database's heart failure population may have biased our results. However, we must also emphasize that the factors successfully screened through various statistical methods have positive significance. Additionally, limited by published data, our study did not classify heart failure but rather studied multifactorial heart failure, which limits the elevation of our study's significance. Furthermore, as Mendelian randomization has inherent methodological limitations, our study is subject to issues such as data heterogeneity across different datasets and potential pleiotropy, where genetic variants may influence multiple traits beyond the exposure of interest. This could introduce bias in estimating causal relationships. Moreover, the observational nature of MR analysis means it cannot establish definitive causal mechanisms but only infer associations based on genetic proxies, which is an important limitation of this approach. Lastly, as MR requires that the exposure and outcome populations be from the same race but different groups, our choice of populations for both exposure and outcomes being Europeans makes it difficult to apply our results to other races, presenting another limitation of our study.

## 6 Conclusion

We identified CXCL9, IFN-γ, LIFR, and UPA as potential inflammatory factors associated with heart failure through forward Mendelian randomization. These findings suggest potential therapeutic targets but require further validation.

## Data Availability

The original contributions presented in the study are included in the article/Supplementary Material, further inquiries can be directed to the corresponding author.
