# Supplementary figures and images for: The relationship between inflammatory factors and heart failure: evidence based on bidirectional Mendelian randomization analysis
Source: Front Cardiovasc Med. 2024 Dec 12;11:1378327. doi: 10.3389/fcvm.2024.1378327 (PMC11669679; doi:10.3389/fcvm.2024.1378327)

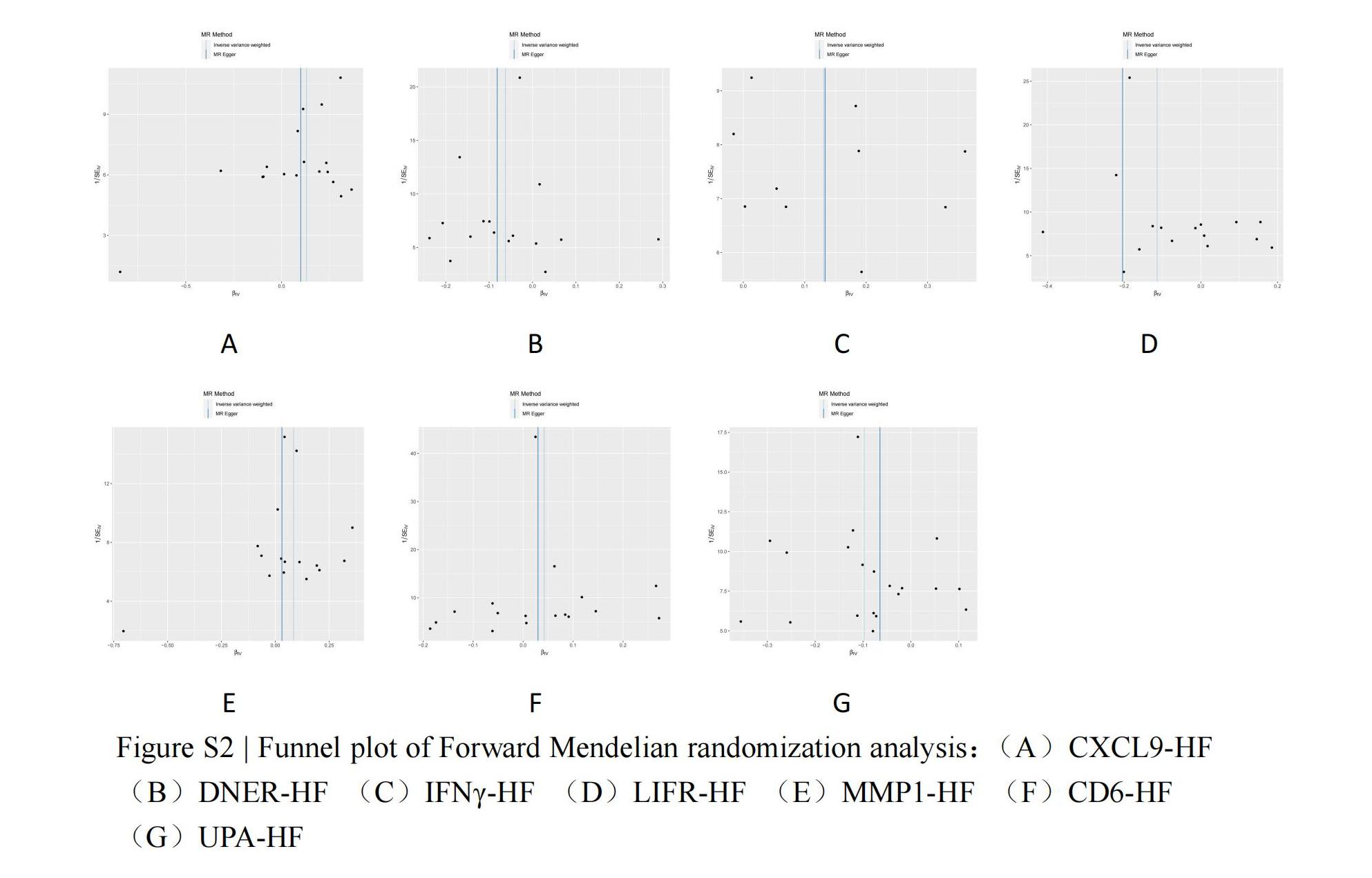

Supplement: Supplementary file 1 [file Datasheet1.zip › FigureS2.jpg]

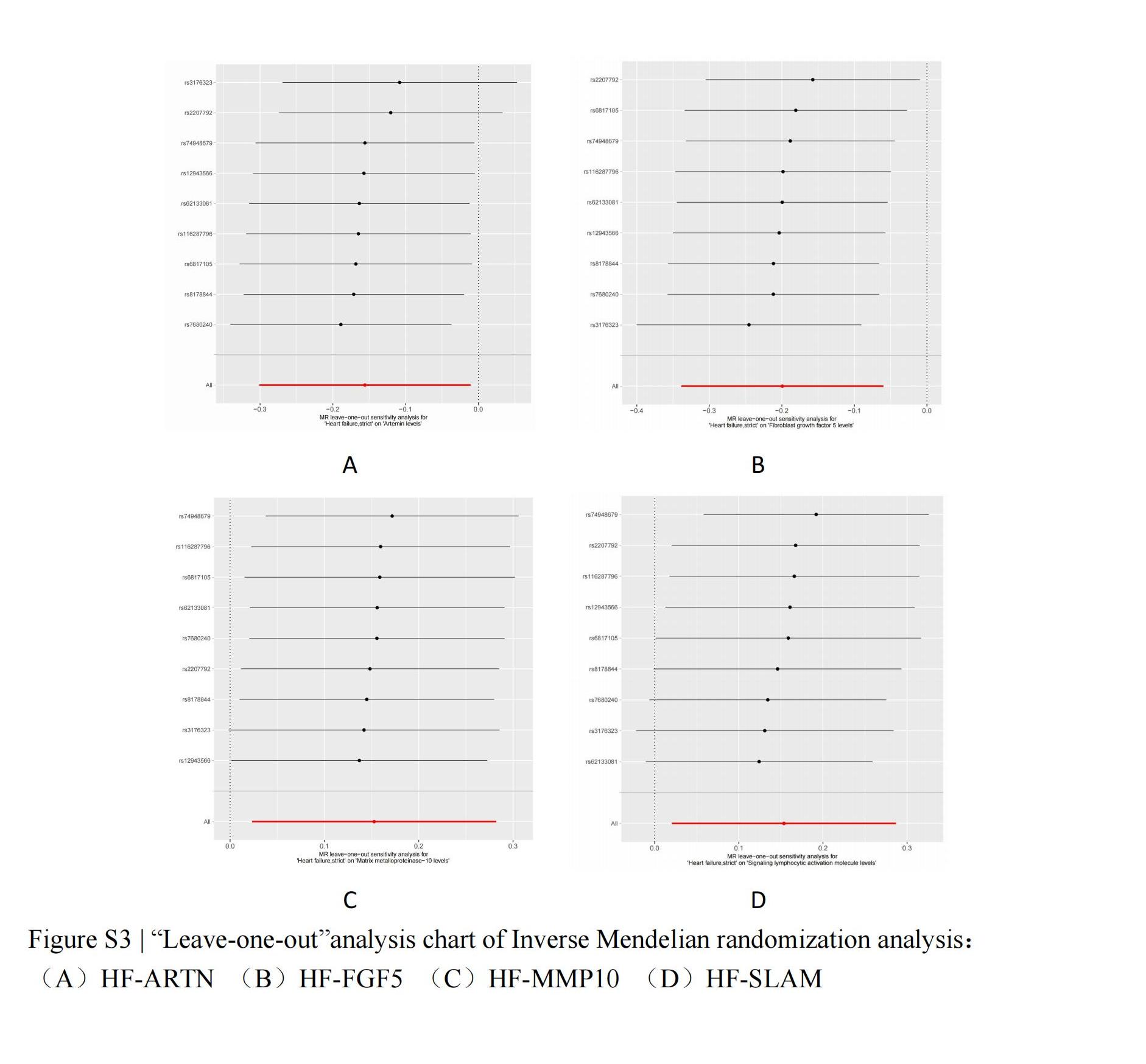

Supplement: Supplementary file 1 [file Datasheet1.zip › FigureS3.jpg]

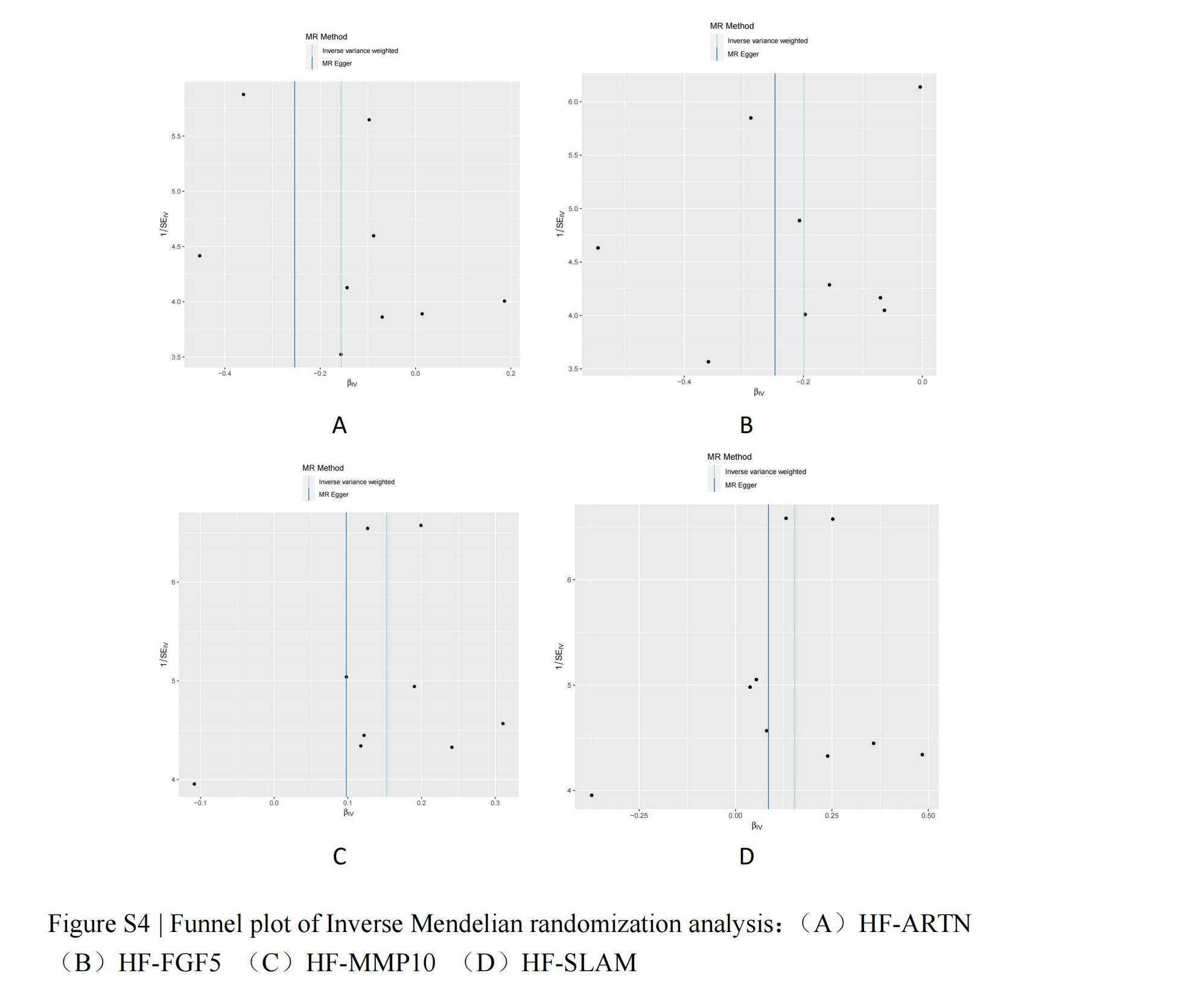

Supplement: Supplementary file 1 [file Datasheet1.zip › FigureS4.jpg]

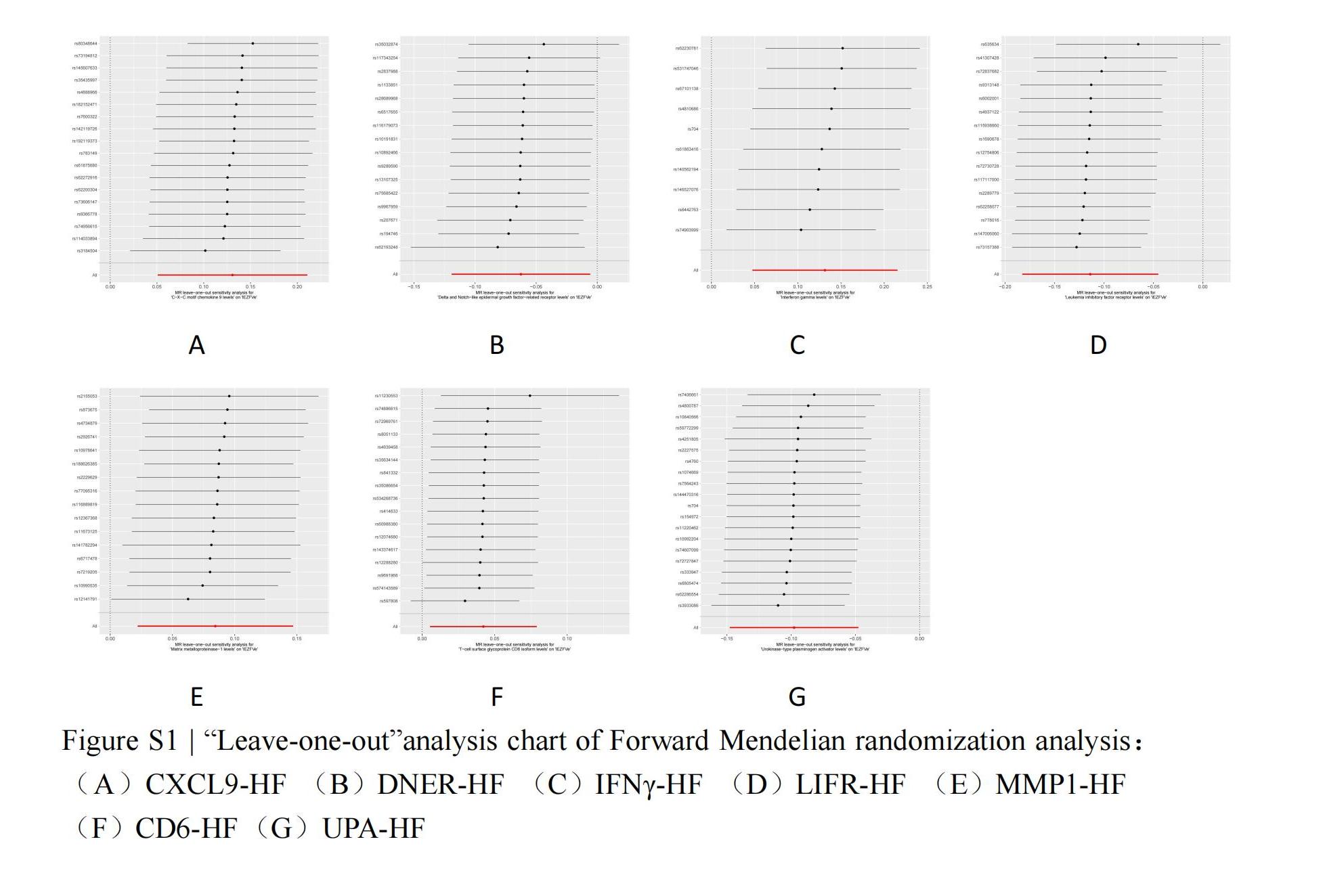

Supplement: Supplementary file 1 [file Datasheet1.zip › FigureS1.jpg]
